# Problematisations of Complexity: On the Notion and Production of Diverse Complexities in Healthcare Interventions and Evaluations

**DOI:** 10.1080/09505431.2016.1212003

**Published:** 2016-09-19

**Authors:** Tineke Broer, Roland Bal, Martyn Pickersgill

**Affiliations:** ^a^ Usher Institute for Population Health Sciences and Informatics, University of Edinburgh, Edinburgh, UK; ^b^ Institute of Health Policy and Management, Erasmus University Rotterdam, Rotterdam, The Netherlands

**Keywords:** quality improvement programmes, evaluation studies, complexity, health care, design, The Netherlands

## Abstract

Within the literature on the evaluation of health (policy) interventions, complexity is a much-debated issue. In particular, many claim that so-called ‘complex interventions’ pose different challenges to evaluation studies than apparently ‘simple interventions’ do. Distinct ways of doing evaluation entail particular ontologies and epistemologies of complexity. They differ in terms of whether they define complexity as a quantitative trait of interventions, whether they see evaluation as part of or outside the intervention, and whether complexity can be regarded as an emergent property of the intervention and its evaluation. In practice, evaluators and commissioners of large health care improvement programmes rely on different, sometimes contradictory, repertoires about what it means to conduct a ‘good’ evaluation. This is an ongoing matter negotiated between and among commissioners, researchers, and—sometimes—programme managers. In particular, notions of evaluability, usefulness and distance/independence are problematised in different ways and with diverse consequences, which, in turn, produce other notions and layers of complexity such as temporal, institutional and affective complexities. When (social science) researchers claim that one method or another is better able to grasp complexity, they elide the issue that any methodological choice emphasises some complexities and lets others fade into the background. Analysing the practicalities and emotions involved in evaluation studies opens up the notion of complexity to analytical scrutiny, and suggests a basis for co-theorising between biomedical, public health and social scientists (including Science and Technology Studies scholars).

## Introduction

Within the literature on the evaluation of health (policy) interventions, complexity is a much-debated issue. In particular, many authors claim that so-called ‘complex interventions’ pose different challenges to evaluation studies than apparently ‘simple interventions’ do. In guidelines for conducting randomised controlled trials (RCTs) on complex interventions in health care and public health, the UK’s Medical Research Council (MRC) defined complex interventions as those that address ‘behaviours, parameters of behaviours (e.g., frequency, timing), and methods of organising and delivering those behaviours (e.g., type(s) of practitioner, setting and location)’ (Medical Research Council, 2000, p. 2). While influential, this is but one definition of what complex interventions are. Various authors have sought to define them, and debates continue regarding how the ‘complexity’ of such interventions can be conceptualised. In turn, discussions ensue regarding what such definitions and conceptualisations imply for the conduct of evaluation studies (i.e. studies investigating the implementation, processes and results/effectiveness of the interventions) (cf. Bonell *et al.*, 2013; Marchal *et al.*, 2013).

To elucidate notions of complexity in evaluation literature and practices, we report here on an analysis of literature on evaluating complex interventions and, furthermore, draw on qualitative data regarding the experiences with evaluation studies of six health policy interventions. Recognising and developing the work in this area by scholars such as Cohn *et al*. (2013) and Salisbury *et al*. (2011), we study practical challenges involved in complex interventions and their evaluation, reflecting on the kinds of complexities that are configured in the process. Our research questions are twofold: (1) What (notions of) complexities underlie and are significant for different means of evaluating complex interventions? (2) What issues are salient for actors involved in the evaluation process, and at what point(s)? In addressing these questions, we do not seek to ‘take sides’ in debates about interventions, evaluation and complexity. Rather, we intend to participate in discussions about complexity by elucidating some of the issues and dilemmas that emerge from the literature on and practice of evaluation studies.

In the next two sections, we discuss our analytical perspectives and methods. Then, in the first section of the results, we analyse some of the explicit and implied definitions and uses of ‘complexity’ within the trials and evaluation literature. In doing so, some of the epistemological and normative stances that underpin them are elucidated (and which are sometimes overtly discussed by scholars in this field). Following this, we shift to analysing how complexity figured within the talk and practice of evaluations in discursive arenas distinct from the more ‘hygienic’ world of journal articles. In particular, we discuss three problematisations of issues that are seen as salient in evaluation practices, and discuss how these produce certain forms and layers of complexity. The paper concludes with reflections on the consequences of this analysis for doing complexity in evaluation studies, and what roles Science and Technology Studies (STS) might play in interrogations and articulations of complexity.

## Analytical Perspectives

In analysing complexity, we draw on two different approaches STS scholars have adopted in theorising complexity.^1^ The first comes from Arribas-Ayllon *et al*. (2010), who discursively analyse scientific articles on psychiatric genetics. Arribas-Ayllon and colleagues follow how the word ‘complex’ is employed within this literature and what functions it has. They go on to show how, within the biomedical world of interventions and evaluations, the word ‘complex’ is not only used to describe the phenomenon under examination—it functions too as a sort of ‘plot’ that frames (and accounts for) research. This includes possible failures to properly understand the phenomenon (i.e. because it is ‘complex’), as well as providing hope for future research (Arribas-Ayllon *et al.*, 2010).

Second, we draw on ideas posed by Mol and Law (2002), according to whom a definitional utterance about the nature of complexity is exactly the moment where complexity gets lost. For these authors,

Things add up *and* they don’t. They flow in linear time *and* they don’t. And they exist within a single space *and* escape from it. That which is complex cannot be pinned down. To pin it down is to lose it. (Mol and Law, 2002, p. 20; emphasis in original)

The opacity of this definition of complexity is, perhaps, precisely intended. Mol and Law go on to argue that questions about whether to accept simplifications *or* more complex understandings of, and ways of interacting with, the world should not really be of concern. Rather, it is ‘a matter of determining *which* simplification or simplifications we will attend to and create and, as we do this, of attending to what they foreground and draw our attention to, as well as what they relegate to the background’ (Mol and Law, 2002, p. 20; emphasis in original).

This perspective inspires us to avoid solely describing what models of complex interventions are (deemed) ‘best’, and enjoins us to distinguish between different modes of ‘doing good evaluation’ (cf. Pols, 2003), considering what kinds of complexities different designs and positions (within evaluation literature and practices) emphasise, and which are relegated to the background. Of course, we are all too aware that we do not have a ‘view from nowhere’ (Haraway, 1988), and that our analysis will itself entail certain simplifications and relegations, in part to try and present a coherent story about a diverse set of practices (Pickersgill, 2013).^2^ We draw here too on the work of Fitzgerald and Callard (2014; see, relatedly, Fitzgerald *et al.*, 2014). They call for an experimental approach to collaboration and co-theorising between, in their case, social scientists and neuroscientists, and we use their approach to think through what it means to co-theorise the notion of complexity with the people involved in healthcare interventions and evaluations.

While taking cues from writers such as Arribas-Ayllon, Mol and Law, and the larger literatures they emerge from and talk to (e.g. Actor Network Theory (Law, 1999)), we also divert from these approaches to complexity in some ways. In contrast to Arribas-Ayllon and colleagues (2010) we are not exclusively following the use of complexity (i.e. treating it as an actors’ category) but we also use it as an analyst’s category, taking seriously the notion that certain situations are indeed ‘complex’, in the sense that they generate practical, cognitive and affective dilemmas for practitioners. In effect, our ontological imaginary is somewhat more performative than that constituting the explicitly constructivist analysis of Arribas-Ayllon *et al.* (2010), which thus places us closer to Mol and Law. In sum, our approach is to interrogate how particular issues arising in evaluation studies and the strategies employed to deal with these issues embody and lead to different notions and layers of complexity.

Following Foucault, social scientists have shown how the way an issue is defined also has consequences for what strategies are chosen to deal with it, with the term ‘problematisation’ used to refer to the twinned process of defining and tackling problems (e.g. Callon, 1986). The notion of problematisation is closely linked to both ontology and epistemology, as many social scientists have shown. In fact, it could be argued that problematisation interweaves the two, as a problematisation combines a problem definition and the strategies used to deal with the problem (or strategies to get to know the problem, i.e. epistemology) (Callon, 1986; Pickersgill, 2011; Neyland and Milyaeva, 2016). For Foucault (as Lemke (2011, p. 32) has argued), problematisation (e.g. of madness or sexuality) is both an actor’s and an analyst’s category: the emergence and conditions of existence of particular problematisations can be studied, but at the same time the observer too engages in this process of problematisation.

In analysing the issues arising in evaluation studies, including the strategies employed to deal with these issues, we draw on the notion of ‘problematisation’. We do so to theorise both the ontological and epistemological notions of complexity, within the literature on evaluation studies of complex interventions and in evaluation practices themselves. In evaluation studies of complex interventions, problematisations of salient issues in evaluation practices produce different versions of complexity that may or may not be similar to those found in the literature on evaluating complex interventions. Through our analysis of complexities within both scientific articles and in practice, we seek to cast fresh light on some of the variety of epistemologies and ontologies of complexity relating to problematisations that occur when scholars debate and evaluate ‘complex interventions’.

## Methods

Our study consists of two parts. First, we examined reports and articles on how to evaluate complex interventions. These involved discussion articles, guideline reports and actual evaluation studies. We attended especially to documents produced by the UK MRC and the US Agency for Healthcare Research and Quality (AHRQ) since these are two dominant groups in the health care evaluation field. From there we snowballed through the literature, exploring also perspectives differing from more ‘traditional’ experimental designs. Our key search terms included ‘evaluation’, ‘evaluation study’ and ‘complex interventions’. We also asked groups researching complex interventions for relevant literature. Moreover, we looked in key journals to search for ongoing debates.

We tried to cover the range of positions possible in the debate on evaluation studies of complex interventions in health care without aiming for a full review within those positions. Each of the evaluation designs implicitly or explicitly holds a view on what complexity means (ontology) and how it can be captured in the best way (epistemology), and ascertaining these perspectives has been our main focus during the analysis: that is, the identification of different ways of constructing and producing ‘complexity’. While most of the reports and articles mention the term ‘complex evaluations’ explicitly, sometimes ‘complexity’ was an implicit notion rather than an explicit one. In those cases, we sought to deduce from the way these articles talked about health care interventions/programmes their stance towards complexity, where complexity can then be seen more as our analyst’s category rather than an actor’s category per se.

### Analysis of Six Evaluation Studies

Secondly, we qualitatively examined evaluation studies associated with six large improvement programmes in different health care settings carried out in the Netherlands between 2005 and 2011. We deliberately chose to focus on rather diverse programmes, in order to capture possible variety in problematisations of complexity: the programmes differed in terms of their size, how many projects were part of them, how they were initiated, and what health care sector(s) they addressed (e.g. hospitals, mental health and nursing homes). The programmes were led by different organisations as well, generally ones specialising in implementing improvement programmes in the specific sector targeted.

The evaluation studies accompanying these programmes also varied, and they were conducted by different (research) organisations. Some of the evaluating teams worked at universities, but others worked in consultancies or research organisations specifically doing research studies for policy (which we refer to in this article, for reasons of clarity and continuity, as academic versus applied evaluators—even though we note that these terms are not mutually exclusive or unproblematic). Most used a mixed methods design, involving both quantitative and qualitative methods, except for one that featured solely a qualitative process evaluation (for an overview of the evaluations, see Øvretveit and Klazinga, 2012). The programmes and evaluation studies were all commissioned by ZonMw, the Netherlands organisation for health research and development. A selection criterion was that the evaluation studies should have been recently finished at the time we started our study, so that we could also incorporate experiences with the final reports of the evaluation studies, how they were received, and how different actors looked back on the evaluation studies.

To investigate experiences with evaluation, interviews were conducted with five project leaders of evaluation studies (working at different sorts of research organisations or at universities) and four study commissioners (all working at ZonMw), as well as with one performer of the interventions. Broer conducted the interviews between July 2011 and April 2012, asking about the respondents’ experiences with the evaluation study, contact with other actors involved, and what they would have (wanted to be) done differently next time. In preparing for the interviews (and in order to contextualise the analysis), Broer read and analysed the reports of the evaluation studies, and, when relevant, adjustments to the topic guide were made to reflect the particularities of the respondent’s context. Generally, however, the topic lists used were the same per interview. In addition to the interviews, Broer and Bal convened a focus group with 12 ZonMw-commissioners of evaluation studies, to explore their experiences with (commissioning) evaluation studies and with aligning the programmes and the accompanying evaluation studies.

Broer and Bal were both involved in one of the evaluation studies that incorporated within the dataset, and these experiences informed the analysis. More specifically, they were involved in an evaluation study of the programme ‘Care for Better’, which focused on long-term health care, such as care in nursing homes and long-term mental health care. The evaluation study was conducted by a larger team, consisting of both quantitative and qualitative researchers. As part of the evaluation study, Broer and Bal met occasionally with the commissioners of the evaluation study, and conducted participant observation of two of these meetings, taking detailed notes that were written up immediately following them. Furthermore, the commissioner of the evaluation study (ZonMw) also organised meetings among researchers and commissioners of all the large improvement programmes that ZonMw commissioned and observation were undertaken of these meetings too. The meetings were held twice a year over a period of five years, and all were observed.

All interviews and parts of the focus group session were recorded with permission, transcribed and coded. Detailed notes were also taken during the focus group. Draft texts of the analysis were then circulated and discussed with both evaluators and commissioners for member check and to get additional input. All respondents consented to the interviews, the focus group and the observations.

In our analysis, we focus on three problematisations of issues that arose for evaluators and commissioners of evaluation studies, highlighting their different perspectives and (how they accounted for) the choices that they made. In the conclusion, we come back to how these different problematisations relate to different kinds and notions of complexity, including those we located within the literature. It is to the different notions of complexity within writings on (evaluation studies of) complex interventions that we now turn.

## Empirical Analysis

### The Complications of Complexity

The MRC is one of the key sponsors of biomedical research in the UK; it is part of, and contributes to, an international tradition in which experimental designs are seen to provide the best evidence for interventions (Timmermans and Berg, 2003). The MRC is a major proponent of complex interventions, producing documents that have had impact far beyond the shores of Great Britain and Northern Ireland (Campbell *et al.*, 2007). Cohn *et al*. (2013) note how this organisation was one of the first funders to address contemporary health care interventions as ‘complex’, described as ‘involving overlapping modes of operation, and hence challenging any straightforward measurement or evaluation of their impact’ (p. 40). They connect this interest in and rise of complex interventions to the need for health services to change social and biological factors simultaneously, relating to a rise in chronic conditions (Cohn *et al*., 2013). While the title of the MRC’s 2000 ‘framework’ referred explicitly to RCT’s, this reference was dropped in the title of its 2008 ‘guidance’ since the organisation was aware of the impossibility or undesirability of RCT’s in some circumstances (MRC, 2008; Cohn *et al.*, 2013). However, even though the MRC acknowledged other possibilities, it still argued in favour of experimental studies, and for RCT’s to be conducted wherever feasible.

Another key advocate for complex interventions is the US AHRQ. Many of the articles published under its aegis draw on a social scientific framework within which the role of theory in evaluating interventions has an important place (Foy *et al.*, 2011). At the same time, the organisation asserts: ‘rigorous experimental or quasi-experimental methods are still required to draw conclusions about effectiveness’ (p. 456). These should be complemented by process evaluations assessing ‘the intended and unintended changes in processes that may affect outcomes’ (Foy *et al.*, 2011, p. 456). Hence, while the AHRQ takes a slightly different approach from the MRC (i.e. it draws on social scientific rather than solely biomedical theory in evaluating interventions), both organisations place an epistemological premium on experimental designs.

In both cases, an understanding of complexity as quantifiable is inherent to the formal articulation of what a complex intervention is. The MRC, for example, defines complex interventions as comprising several countable characteristics: the number of interacting components (i.e. behaviours, variables, outcomes, etc.), the number and complexity of behaviours of actors, the number of target groups for the intervention, the number and variability of trial outcomes and the degree of flexibility in trial design permitted (Craig *et al.*, 2008, p. 979). Hence, the characteristics constituting the degree of complexity can be closely defined and measured. Accordingly, like (Arribas-Ayllon *et al.*, 2010), we argue that here ‘complexity is (viewed as) merely a quantitative increase in “complicatedness”’ (p. 500), rendering it as the (quantifiable) mirror image of ‘simplicity’.

Complex interventions are sometimes also thought of as representing a ‘paradigm-shift: a Kuhnian revolution in which linear and reductionist tools and methods are replaced by non-linear and dynamic counterparts that are capable of grasping complex objects’ (Arribas-Ayllon *et al.*, 2010). The relatively recent development of ‘realist reviews’ as a means of evaluating complex interventions casts light on the degree to which complex interventions are framed as innovative (i.e. since the innovative nature of complex interventions is regarded as necessitating innovation in the means by which they are evaluated). Realist reviews of healthcare interventions have been proposed and developed by a group of UK social policy and healthcare researchers and have been adopted widely (Pawson *et al.*, 2005). Realist review has, according to its proponents, no preference for quantitative or qualitative methods, but, rather, sees ‘merit in multiple methods’ (Pawson *et al.*, 2005, p. 22). Indeed, realist reviews themselves can be considered as part of the wider evidentiary paradigm shift within which complex interventions are often positioned by investigators and commentators.

Realist reviewers have described the realist method as entailing a change in the question that an evaluation addresses: ‘Under realism, the basic evaluative question—what works?—changes to “what is it about this programme that works for whom in what circumstances?”’ (Pawson *et al.*, 2005, p. 22) This more closely specified and contextualised question is understood to refocus evaluative goals from judgement to explanation. In doing so, realist reviewers seek to overcome some of the problems regarded as emerging as a consequence of the application of experimental research methods to ascertain the effects of an intervention (Pawson *et al.*, 2005; Greenhalgh *et al.*, 2009). Advocates argue that ‘large-scale, whole-systems interventions in health care require imaginative approaches to evaluation that go beyond assessing progress against predefined goals and milestones’ (Greenhalgh *et al.*, 2009, p. 391). In realist review, the triplet ‘context, mechanism and outcome’ is often used in order to evaluate an intervention. Indeed, ‘[r]ealist review seeks to uncover the underlying theories that explain these demiregularities [in which human beings generally make similar choices in relatively similar contexts] by critically scrutinising the interaction between context, mechanism and outcome’ (Wong *et al.*, 2010, p. 15). Furthermore, by asking ‘what works for whom’, realist reviewers take seriously the views and practices of practitioners in defining and dealing with complexity in specific programmes (Greenhalgh *et al*., 2009).

In the process of undertaking a realist review, seven defining features of complex interventions are outlined, including that they work through the choices of stakeholders, have non-linear implementation chains, and are shaped according to specific contexts (Pawson *et al.*, 2005). By addressing these features, realist evaluation seeks to produce rich descriptions of the different mechanisms leading to change without assuming a linear and causal relationship between the mechanism and (positive) outcomes. In a sense, it is precisely the inability to quantify all relevant factors in an intervention that seems to constitute the ‘complexity’ here. Moreover, as realist evaluators suggest that the nature of the intervention only becomes clear in its development, the (number of) components to it can only be settled retrospectively.

Others argue that evaluation itself is an element that can make interventions complex. Within this proposition, which in fact pre-dates the idiom of complex interventions, an understanding of evaluation as a neutral entity positioned outside the intervention is refused. Authors such as Guba and Lincoln (1989) and Abma (2005, 2006) frequently criticise overtly instrumentalised forms of evaluation, especially when undertaken at the behest of policy-makers. By aligning with policy objectives and evaluating a programme in terms of whether these are met, the truth (and the complexity) of the intervention is taken to be defined in a singular way—other means of understanding and speaking about ‘effectiveness’, for instance, are deleted.

Based on this criticism, different sorts of evaluation studies have been increasingly employed since the late 1980s, such as fourth generation evaluation (Guba and Lincoln, 1989) and responsive evaluation (Abma, 2005, 2006). While such approaches that resist instrumental uses (and, more generally, a strictly positivistic view of knowledge production) have been used in evaluation studies of health care interventions (as we will outline below), they are especially common in the field of education (where there has traditionally been more room for approaches other than experimental ones; Oakley, 2006). These evaluative studies share a constructionist perspective, and evaluators investigate what the intervention means for, among others, the clinicians and patients involved and how they did (or did not) use it. The underlying rationale is that evaluators do not put forward their own truths, but that facts are instead produced through dialogue between the different actors engaged in a programme and its evaluation (Guba and Lincoln, 1989; Mabry, 2002; Abma, 2005, 2006). Citizens (or, in health care, patients, among others) are framed as relevant stakeholders and participate in the evaluation, such as by contributing to its organising questions, helping to select participants, and working with academics in the interpretation of findings (Guba and Lincoln, 1989). The complexity at stake here appears to be a property of the intertwinement between the intervention and its evaluation, and the multiplicity of meanings, goals and truths inherent within and constituted through the evaluation assemblage.

Authors proposing so-called ‘formative evaluations’ seem to take the issue of evaluators contributing to the intervention even more seriously. Implicitly, many of the designs described above make a distinction between research and policy: research is meant to inform policy-makers, or is understood as being appropriate to conduct in isolation from political/policy goals. Another way in which evaluation can be conceptualised, however, is as *part* of policy-making. Formative evaluations—that is, evaluations that are formative to the interventions studied as they are performed—are undertaken with this interpolation as an explicit organising rationale.

As such, formative evaluations do not draw rigid boundaries between ‘interventions’ and ‘research’. In more traditional experimental designs, intervening in the eventual implementation might be problematic since the more the evaluation impacts the intervention, the less valid the conclusions might be (Bate and Robert, 2002). Within formative evaluations, on the other hand, it is argued that, first, evaluation cannot be (entirely) distinct from the intervention it evaluates, since the evaluation will always have a certain impact, and, second, evaluation should ideally contribute to the intervention programme along the way since it would be ‘ironic’ to carry out a ‘non-learning evaluation of a learning programme’ (Bate and Robert, 2002, p. 974). Accordingly, proponents plea for formative evaluations within which the emerging results of the evaluation are used immediately for informing programme change (Øvretveit, 2009; Zuiderent-Jerak *et al.*, 2009). It appears that ‘complexity’ in formative evaluation can be understood as emerging from the ambiguous and intertwining ontologies of ‘policy’, ‘intervention’ and ‘research’, and the performative approach to evaluation necessitated by this comprehension of experimentality (cf. Pickering, 2002, 2010).

As we have seen, a range of understandings of complexity inhere within, associate with, and are articulated and produced through biomedical and social science discourses on so-called ‘complex interventions’ and their evaluations. These differ in terms of whether they define complexity as a quantitative trait of interventions, whether they see evaluation as part of or outside the intervention, and whether complexity can be regarded as an emergent property. In the next section, we analyse experiences with evaluation studies of six different improvement programmes (which are generally seen as complex interventions) and, in particular, describe three problematisations in evaluating complex interventions. We then reflect on the similarities and differences between accounts in the literature and accounts from our interviews and what those mean for notions of complexity. Our analysis treats the different problematisations in terms of their conceptualisation and constitution of complexity within evaluation practices. Each problematisation does not relate to one particular form of complexity as such; rather, the specific ways in which problems get defined and dealt with respond to and produce different kinds of complexities.

### Problematising Evaluability

As described in the methods section, we studied six evaluation studies of large ‘quality improvement programmes’ in the Netherlands. While these programmes were not generally referred to as ‘complex interventions’, this term was frequently mentioned in meetings between evaluators and commissioners, and the architecture of the programmes corresponded with existing accounts of complex interventions within the literature. The programmes were the first of their kind in the Netherlands, and both evaluators and commissioners struggled with coming to grips with this new ‘beast’ (as some evaluators sometimes referred to the programmes). The improvement programmes were nation-wide activities in which up to hundreds of care organisations and teams participated, focused on several processes (or subjects that were deemed in need of improvement, such as medication safety, fall prevention, etc.) in the organisations and at different organisational ‘layers’. The programmes were also multifaceted in terms of the ‘interventions’ employed, with guidelines, consensus meetings, websites, advisors to the organisations, national teams and many more part of the overall programmes.

As we have seen, evaluation is a key component in complex interventions; moreover, it is generally regarded as something that can be done *well* (or badly). Accordingly, perhaps the most significant problematisation that we deduced orientated around the question of how to make a programme ‘evaluable’. People involved in evaluation studies of improvement programmes problematised this issue in different ways, with various strategies discussed and deployed to deal with it. While to some extent such strategies could be thought through at the start of the evaluation, this matter seemed to be an ongoing evaluation question as well, since study design is but one factor determining the strategies employed to make the programmes ‘evaluable’. Hence, the problematisation of evaluability happened throughout the duration of evaluation studies.

One key aspect of making programmes evaluable concerned their goals. Complex interventions, including the quality improvement programmes under consideration here, are designed to meet specific goals, which are often framed as ‘SMART’: Specific, Measurable, Actionable/Ambitious, Realistic and Timely. Goals serve a double function: they are meant to encourage organisations to participate and to be ambitious in their projects, and to convince policy-makers of investing in them; also, they are used in project evaluations to judge the success of a programme. Tensions can arise between the need for outward-facing bold and ambitious goals, and a practical imperative for more mundane and easily measurable targets that will help the programme to be evaluated (and to do well in the evaluation).

For the interventions and evaluations we examined, the usual strategy was to set ambitious goals, but then ‘one builds in disappointment’, as a senior ZonMw employee argued. Because goals were set that would excite people and attract organisations to the quality improvement programme, it meant that goals were sometimes less realistic—and ultimately seldom met. Generally, the evaluations concluded that such programmes were not as successful as expected.

In order to meet at least the majority of the goals, some ZonMw employees moreover asserted that these should have been made more explicit from the programme conception—echoing, therefore, the positions on evaluations set out by the MRC (2008, p. 12) and AHRQ (cf. Foy *et al.*, 2011). As one interviewee working for ZonMw argued:

I think it is very important to think about the criteria for evaluation when starting the programme. What aspects of the programme do you want to have evaluated? And that also means that one should think about the programme goals. What do you want to achieve? Because that is what you want to be evaluated on. ( … ) In contrast to how it was mostly done: that we thought about the criteria for evaluation halfway or towards the end of the programme.^3^ (Senior programme manager, ZonMw)

Some of the evaluation studies indeed defined the goals and indicators for improvement beforehand, and measured programme success on the basis of these, aligning with the wishes of the ZonMw employee quoted above. However, both evaluators and programme managers alike were hesitant about this strategy of defining success on the basis of these predefined goals. One programme manager—who led the overall programme ‘Care for Better’ of which Broer and Bal acted as evaluators—was critical about the use of these goals for the evaluation:

Well, I understand, you need to know: if you set a goal, is it met. But the setting of the goal for us was a little … [makes a gesture with her hands to indicate they were not thought through specifically for evaluation purposes] If that then becomes such an important hook for the entire project. Setting a goal is very difficult and when these [goals] then become what you will be judged on in the end—that’s how it feels sometimes—then that’s not justified. It makes me think: what is the value of this conclusion and is it actually correct? […] Also because the people [who are setting the goal] were very ambitious at that time and wanted to get the funding for the project, so how realistic is it [the goals]? (Senior Consultant/Researcher)

In other evaluation studies defining goals and indicators beforehand was seen as a problem to start with, or even as not doable, because programmes and goals often change during implementation. The programmes can become bigger, with parts added; for example, the programme Broer and Bal evaluated began with seven improvement projects but soon increased both the number of projects and the sectors addressed (i.e. mental health care, in addition to the care of older adults). Such expansions can create difficulties and frustrations for evaluators. An evaluator of a different programme said:

I am never going to do that again (…) [i.e. evaluate a programme that keeps changing]. It has to be clear what the evaluation has to be, and it should not be the case that we are submitting a proposal for a train that is already moving and where new elements are constantly added to, so that we need to develop new questionnaires and interviews and so on. (Head of Department, Research organisation)

Further, teams in healthcare organisations performing the interventions might have started working on some goals—yet, they later realised that they needed to work on something else. An example here, of the evaluation Bal was involved in, is of an improvement team that originally had the goal of decreasing the number of reported safety errors (medication safety, falls, etc.), but they soon decided that the ‘real’ problem was that most errors are not reported in the first place. Accordingly, the goal changed to increasing the number of reported errors (Zuiderent-Jerak *et al.*, 2009). In contrast to the conventional logic of evaluation, some researchers argued that programme goals should not be fixed beforehand, but should be, to an extent, emergent. Such assertions resonated with some of the literature on fourth generation evaluation discussed in the previous section (Guba and Lincoln, 1989). At the very least, the respondents considered, there should be facility for shifts and displacements in goals, and an evaluation framework that allows for this should be employed.

In this respect, we detected two slightly different strategies that evaluators opted for, sometimes even within the same evaluation. In some programmes, the improvements on those goals the improvement teams themselves had set were used by evaluators as measures of success, rather than or in addition to goals the evaluators had predefined. As part of this strategy of staying close to what improvement teams were doing, some researchers also used process evaluations to describe and explain these goal displacements. As a second strategy, outcome measures such as perceived effectiveness were also regarded as a way of enabling teams to measure specific goals while still allowing for the evaluation of their efforts on predefined measures. Hence, teams within the programmes were still working on the issues that they found important while evaluators ensured that the programmes were still ‘evaluable’—that is, that at least something was being aimed towards, and that any progress made was measured.

Goal displacement not only needs flexibility on the part of researchers, however, but also from research commissioners: study questions and methods might change, so the knowledge ultimately produced might not accord with that initially commissioned. As an organisation connecting research and policy, ZonMw (i.e. the commissioner) itself has a range of aims, such as enhancing knowledge, improving healthcare and connecting with government agendas. Reflecting on ZonMw’s goals, one ZonMw employee mentioned that evaluation research can be seen as an evaluation of the work of ZonMw itself:

the simplest thing is that we want to evaluate our own work: have we done it right? Have we done the right things and have we allocated the money in the right way? Because we want the money to be spent in the right way to actually contribute to Dutch health care. (Head of Department, ZonMW)

This will towards institutional reflexivity—so characteristic of many private and public bodies today (Giddens, 1991; Power, 1997; Dahler-Larsen, 2012)—existed alongside other, sometimes more traditionally instrumental goals and expectations. These included acquiring knowledge that the project researchers and ZonMw could use to: (a) immediately improve the programme (as per some of the literature on evaluation interrogated in the previous section); (b) draw conclusions that could be taken into account in future decisions and programmes (a classic motivation underlying evaluation) and (c) understand better what the programme had achieved (for the immediate sake of the organisation, as well as to show others what societal benefits public investment in it has afforded). Yet, although these different and sometimes contradicting aims of ZonMw could lead to tensions both within and between evaluators and programme managers, they also had the potential to give leeway for both sets of actors to pursue their own paths.

In sum, making programmes evaluable entails goal setting and finding suitable evaluation methods, and different ways of problematising evaluability of a programme come with different ontologies and epistemologies of complexity (e.g. questions come to asked regarding whether programmes should be seen as predefined or emerging, with implications for how these different ontologies should be captured in the evaluations). This is an ongoing process that requires flexibility on the part of evaluators, commissioners and programme managers alike (and even then can involve friction). The setting of goals sometimes creates ambivalence between programmes and evaluations, as goals might have different meanings and functions within these two practices, and must also resonate in some way with the overarching (and varied) aims of ZonMw. The problematisation of how to produce ‘an evaluable programme’ also entails and exhibits specific complexities, such as practical, political and affective notions of complexity. Only through grappling with the practicalities can any epistemological purchase be found—the practicalities interact with, rather than merely follow from, different kinds of complexity.

Emotions in this respect played a large role in how evaluations are practiced: frustration, anger, unease and hope that it will all work out in the end. What, when and how tensions and emotions came to the fore depended on the specific evaluation strategy (and epistemological position) adopted. In more ‘traditional’, MRC-style approaches based predominantly on a quantification of complexity, the programmes were seen as ‘moving targets’ and programme executors and evaluators had to come to agreement over goals. Conversely, in more interventionist approaches there was a constant tension (and ensuing epistemic and emotional labour) in trying to define the precise goals of the programmes while remaining accountable to outsiders/policy-makers. Our analysis here shows some resonances with the kinds of emotions activated in the interdisciplinary work documented by Fitzgerald and colleagues (2014), where diverse agendas and expertise are likewise brought together—sometimes antagonistically—in order to produce knowledge.

### Problematising Usefulness

Another important issue for commissioners and researchers was how to produce ‘useful’ knowledge. For one thing, it was not obvious what ‘useful’ might mean for the different actors involved. Roughly, among the evaluation studies we investigated, a distinction can be made between first, those conducted by more ‘applied’ evaluators, whose aim is to improve policy and services, and second, evaluations carried out by academic research organisations. These distinctions are not always clear-cut, however; for instance, academic evaluators can also have a practice-orientated focus. Nevertheless, the former group usually gives fairly straightforward answers to questions regarding what is needed from an evaluation project and for whom. Further, unlike academic researchers, their work is not explicitly aimed at generating generalisable knowledge and rarely leads to (international) publications.

As a consequence of its lack of ‘application’, the value of academically-orientated evaluation was sometimes difficult to see from a ZonMw perspective. As one senior ZonMw employee asked, rhetorically: ‘What do we benefit from an article in the Journal of Anthropology?’ This illustrates a tension between two different modes of ‘doing good evaluation’ (cf. Pols, 2003), and the diverging epistemological and ontological biases, traditions and aims of different evaluators. The more ‘applied’ evaluators seemed to align more closely with the goals of ZonMw; for example, during a discussion at a meeting among commissioners and our academic evaluation team, one of the commissioners sighed to us: ‘You are making *everything* complex.’ Hence, ZonMw more frequently invited ‘applied’ research groups to conduct evaluations as those researchers are more inclined to translate the evaluations into instrumental use. Yet, ZonMw was not *against* international publications; in fact, these two different modes of doing good evaluation were both described by ZonMw employees themselves.

Accordingly, ZonMw could be seen as conceptualising itself as a ‘boundary organisation’, linking academic research and policy programme, and therefore as an embodiment of the possible tensions that could arise between knowledge production and application (Wehrens *et al*., 2014). Some ZonMw employees said that it was often necessary to translate findings from the evaluation for policy and for the general public. For instance, in one of the programmes we were involved in, the programme committee (comprising commissioners, healthcare managers and policy actors) wanted to have 10 bullet-pointed ‘lessons learnt’ from the programme, whereas we did not feel able to translate our data into articulable lessons in the way desired. This was not just an argument about what we (as evaluators) could do, but also about how responsibilities should be organised: we indeed gave presentations with practical suggestions, but saw ZonMw as being primarily responsible for translating this applicable knowledge further. Another evaluator said that over the course of their evaluation they started writing more readable and shorter reports, as was requested also by the programme committee who wanted the reports to be more readily usable for them.

Such examples again illustrate how some evaluators differed from commissioners (and policy actors) in terms of epistemological agendas and ontological frameworks for comprehending the nature of data, and hence the possibility of its purification (and the degree to which this could occur), enmeshed with a disagreement about where responsibilities should and can lie. Bringing in ‘applied’ evaluators represented a (partial) solution for ZonMw, yet also compromised their research goals where programmes were not just set up for implementation but also to produce new knowledge (since ‘applied’ evaluators generally do not focus on undertaking generalisable research). Hence, the production of useful knowledge is more complicated—more complex—than just a straightforward and instrumental matter of ascertaining whether or not the intervention ‘works’ and how the results were obtained.

Usefulness also had another angle. ZonMw respondents, for example, argued that evaluation studies could be more useful when they took into account a programme’s historical and policy context (an aspect which was, according to them, usually absent in the evaluations). Assertions regarding the import of context resonate with arguments advanced by realist evaluators: that is, since the programmes are initiated from and are part of a policy context, this should be taken into account in order to understand the programme properly and to be able to make statements about the programme theories and underlying mechanisms (Pawson and Tilley, 1997). One senior ZonMw employee reflected about an evaluation: ‘If we would have done it [designing the programme] in a different way, would it have had different effects? We don’t know.’ The programmes differed in how they were set up and where the responsibility for improvements was situated, as well as in terms of different policy and healthcare contexts; for at least some in ZonMw, these differences were deemed relevant to understand in order to ascertain how they might lead to variations in outcome (and, if so, what constituted the best set-up of a programme).

Policy contexts, however, are not static; they shift, change, evolve—they are hard to pin down. They are ‘complex’. Given the temporal dimension to policy contexts, the extent to which research is ‘useful’ relates also to its timing. Many researchers note that the results of their work are only partly used by policy actors, if at all. This was perceived to be a consequence of the fact that when the results become available, policy debates and goals have moved on, and/or new programmes have started that do not take the results of the on-going evaluation into account (see also: Øvretveit and Klazinga, 2012; Ettelt *et al.*, 2015). The role of ZonMw is, ostensibly, to mediate between policy and research (cf. Lomas, 2000), but given the long duration of programmes—often four to six years—this would require considerable consistency in the policy domain (which is unlikely).

For instance, in one of the programmes studying new financial instruments to stimulate integrated care, the specific intervention under evaluation was already implemented on a national scale while the evaluation of the pilot was still underway. In the study Broer and Bal were involved in, it was found that the healthcare domain in which the programme was implemented was so crowded with new programmes and initiatives that it was hard to keep up with policy developments. And while ZonMw would make sure that policy-makers from the Ministry of Health would participate in programme committees, and ZonMw leadership had regular meetings with the Ministry as well, such attempts at coordination were certainly not always successful. Hence, the usefulness of knowledge depends on making different time-frames and contexts (of policy, of evaluation studies, of healthcare) align as well.

To summarise, like the construction of ‘evaluable’ programmes, ensuring that programmes produce ‘useful’ knowledge is no simple matter. In particular, questions must be negotiated regarding useful to whom, and for what purposes, with consequences for ontologies and epistemologies of complexity (i.e. the way ‘usefulness’ is defined also interacts with the way an evaluation is seen and carried out, and thus with how ‘complex interventions’ themselves are perceived and produced). Further, the different temporalities of policy, research and evaluations can limit the usefulness of knowledge generated. This especially is an issue that neither evaluators nor commissioners are readily able to negotiate or plan for. The overall effect is to produce a kind of knowledge that has utility within a given timeframe, but also in a highly specific, largely undefinable, and hence inherently complex context—and as the policy world continues to change this usefulness is disempowered.

One problematisation of usefulness occurs, in part, through ZonMw’s preference for ‘applied’ evaluation—yet even these evaluators can falter when programme goals (from which utility emerges) include the production of generalisable knowledge. Moreover, they are powerless in the face of the unpredictability of political decision-making that can swiftly alter the salient features of the context within which they are situated. These different problematisations of usefulness then involve different strategies in terms of aligning with the programmes, of allowing flexibility, or refusing such flexibility as that would hamper knowledge production related to predefined goals or the need to account for ZonMw investments. Such strategies also produced, and were part of a solution for, different notions and layers of complexity, for example, temporal, contextual and institutional complexities.

### Problematising Distance

The final problematisation emerging from our data relates to how involved or distant evaluators should be in the programmes they are evaluating. Independence is often deemed important (though not always possible), both from an evaluators’ and a policy perspective, and can be situated towards different actors and domains. First, the research can be more or less independent from ZonMw. For ZonMw, this is a crucial issue. ZonMw is not a neutral actor; at the very least, it has a stake in the evaluation. One of the elements within the evaluation is whether or not ZonMw commissioned the programmes and evaluation studies appropriately and thus made the ‘right’ choices to improve Dutch healthcare, as one ZonMw expressed it. This made it hard for ZonMw to then comment on an evaluation. As the same ZonMw employee argued:

We can have the appearances against us, you know, when we say this [evaluation] really ‘sucks’, because the researchers can use this against us and say ‘We have evaluated ZonMw and they put our evaluation aside’. That is a real dilemma. (Head of Department, ZonMW)

Second, independence can relate to the connections between the improvement practices and programme executers; here, independence is sometimes argued for—and sometimes warned against. An illustrative example comes from one of the programmes in which so-called ‘guiding research’ took place instead of a formal evaluation. The idea was that this research immediately *served* the programme instead of only *evaluating* it; for instance, feedback was given after people involved in the programme were interviewed, and suggestions made regarding how the programme could be improved. Thus, in this type of research, ‘closeness’ to practice is considered important, with evaluators regarded as having roles to play in the improvement processes.

In contrast, some researchers pleaded for more ‘distant’ research. One senior health services researcher and head of a research department ‘thought that you somehow have to separate evaluation from implementation’, and argued that not having such a (clear) separation might sometimes be detrimental to those very improvement practices. In this evaluation study, intermediate reports were written to conclude the year before and to enable the people involved to use the findings for the next year—which would constitute a way of giving feedback and thus a form of formative evaluation. However, the evaluators’ intermediate reports were not always as positive as programme managers regarded the results/programme to be. The evaluator we interviewed attributed the difference of opinion to programme managers’ strategic use of affirmative tales of the programme as a means of keeping participants motivated. She wondered whether the intermediate evaluation report would harm this strategy, and therefore questioned whether or not it was such a good idea to work with intermediate reports. Hence, the complexities of individual and intra-group psychology (including emotions like excitement and disappointment) were understood to shape the effectiveness of different models of evaluation.

Another example from the same evaluation likewise foregrounded how particular affects could have practical effects. Here, the evaluator had a discussion with the programme management group about what to do with improvement teams that participated in the programme but for which no data was available. According to the evaluators, not taking into account these teams would have led to inaccurate conclusions (in this case, a higher success rate than reflected in the actual programme) and therefore they argued for including these teams in the analysis. The evaluator argued that if the evaluation team had been more involved, they would have faced difficulties in pursuing their argument (i.e. they would have felt personally reluctant) of also taking into account teams for which no data was available. Otherwise, she argued, ‘one would perhaps make other decisions that are more in the interest of the people involved in [the management of] the programme’. In effect, distance resulted in objectivity: proximity might produce personal closeness, regarded here as having the potential to compromise the rigour of the evaluation.

However, not all our respondents agreed with the conclusion that proximity led to personal closeness, resulting in compromised objectivity. One researcher argued that the more independent the research is, the more evaluators needed to work on the relationships that they have with other parties in order to be clear about their role, what they do, and what they do not do:

It is something that should be constantly on your mind. Independence in relation to political parties and in relation to the care organisations; independence plays a role on all levels. (…) Therefore it is important to be constantly aware of it (…) by being very clear to others what your role is (…). Always looking for the dialogue. (Senior Researcher, Research organisation)

In this case, it was felt that by keeping close contact with all involved, enough independence could be created to give a critical analysis of the results. Rather than being distant or involved, a combination of the two is required, needing much relationship work.

We can see, then, that in the same way as complexity is resistant to being understood in practice as a binary issue, the question of whether evaluators should be ‘distant’ or ‘involved’ in the programmes they are evaluating likewise seems to resist a straightforward answer.^4^ The ontologies and normativities of ‘distance’ and ‘involvement’ hence seem themselves to be ‘complex’. Accordingly, the question that occupies evaluators relates to *how* distant and/or involved they should be—as well as *when* and with regards to *whom* and *what*. As with the other problematisations, there are structural constraints operating to pre-discipline the decision-making of evaluators, in ways that in some senses make this challenge easier to deal with: for example, whether or not the evaluation is ‘supposed’ to be (i.e. commissioned as) formative.

Yet, no matter how rigidly defined by commissioners evaluations are (and indeed, there is not often a great deal of rigidity), evaluators still have considerable interpretative flexibility (Pinch and Bijker, 1984) regarding to how they problematise their involvement in and independence to the programme which is interwoven with different ontologies and epistemologies of complexity. These problematisations are contingent, mutable through practice, and relate too to their personal experiences of working with programme teams and to their understandings of the epistemic effects of personal interactions as well as to how different actors see and enact the different responsibilities involved. Again, as in previous sections analysing evaluation practices, such personal interactions and emotions, as well as the different temporalities involved, add new forms and layers of complexities to the complex interventions and their evaluations, such as personal, affective and temporal complexities.

## Conclusion

In this paper, we reported on a twofold exercise: we conducted an analysis of documents writing about (conducting) evaluation studies of complex interventions, and we analysed six evaluation studies to investigate the issues and dilemmas encountered there. Regarding the documentary analysis, we asked: What (notions of) complexities underlie and are significant for different means of evaluating complex interventions? We evidenced different ways of approaching and performing complexity. Evaluation studies can see complexity in terms of quantifying information, such as the MRC-framework tends to do; they can regard complexity as that which escapes quantities such as in realist reviews; or they see evaluations as intrinsically political, and thus as part of the intervention itself, in which complexity emerges as the outcome of this entanglement of intervention and evaluation (and the wider political/policy context) and ‘complexification’ might itself be a research strategy.

These different forms of complexity appeared too in our study of actual evaluation practices. This analysis also addressed the matter of what varieties of complexity seem to structure and be salient to different modes of evaluation, as well as speaking directly to our second research question: What issues are salient for actors involved in the evaluation process, and at what point(s)? People involved in commissioning and conducting evaluations seem to orientate their discourse and actions in ways that involve engagement with (including acts to limit) all these forms of complexity, no matter the design they chose. Different strategies were employed by our various participants in terms of the strategies they employed to deal with specific issues. To varying degrees, evaluators and commissioners seemed to simultaneously rely on different, sometimes contradicting, repertoires about what it means to conduct a ‘good’ evaluation, mixing together different design elements. While the design was to some extent decided upon at the start of an evaluation study, the discussion of how to evaluate a programme was ongoing, between and among commissioners and researchers and, at times, programme managers (in part as a consequence of what was happening in the programmes).

Precisely because of the ongoing and changeable nature of improvement programmes and evaluation studies, we used the Foucauldian notion of problematisation to investigate the way people involved in evaluation studies defined specific problems and solutions to certain issues throughout the study. In particular, they problematised evaluability, usefulness and distance/independence in different ways and with diverse consequences. These problematisations were interwoven with ontological and epistemological notions of complexity, as all problematisations implicitly or explicitly addressed what complexity is in (evaluating) complex interventions and how to best capture or deal with this. In turn, these problematisations produced other notions and layers of complexity, in addition to those complexities locatable within the literature (either by the authors, or by us). In particular, conflicting goals, emotions and temporalities constituted new forms of complexity that had to be addressed in evaluation studies, in which evaluators, programme managers and commissioners all played different roles. These included temporal, institutional and affective complexities.

While we do not suggest that the complexities documented here are necessarily generalisable to other evaluation studies of complex interventions, or that there are no other notions and layers of complexity at stake in such evaluation studies, we hope that our analysis opens up the concept of complexity to more detailed analysis. As well, assuming an evaluator’s ontological and epistemological positions solely on the basis of what design they choose—which might be done when solely reading evaluation literature, and refraining from communication with evaluators, as many scholars writing about evaluations of complex interventions have done (see for an exception: Salisbury *et al.*, 2011)—is problematic, in terms of how social scientists then represent this community, and seek to intervene in their debates and practice (see, relatedly, Broer and Pickersgill, 2015). There is, in effect, the risk of a kind of methodological determinism (Law, 2008). Indeed, we would suggest that in the evaluation literature itself there is a need to deal more candidly and seriously with the practical experiences of evaluators.

Let us continue in this more normative register. Both in the literature we analysed and in the actual evaluation studies we investigated, effectiveness is still predominantly defined in quantitative terms, related to experimental designs (cf. Schouten *et al*., 2008). Qualitative methods give insight into *how* interventions work, but when it comes to conclusive evidence concerning *whether* they work, most authors argue that evaluators need to use experimental designs. However, this is a rather narrow way of defining effectiveness. Evaluation methods like realist evaluation and formative evaluations may provide insight into what the outcomes of an intervention are for various groups, and may also play a role in redefining effectiveness (Zuiderent-Jerak *et al.*, 2009). This, however, can only be done when complexity is not reduced to only that which can be quantified, as for example the MRC tends to do.

That is not to say that quantification in itself is unwarranted, or does not sufficiently address the complexities of the programmes. Quantification can attune researchers to different kinds of complexities that would not be taken into account in more qualitative analyses. When realist evaluators or other social science researchers claim that one method or another is better able to grasp complexity (Cohn *et al.*, 2013), they elide the possibility that making a choice to use one paradigm over another emphasises some complexities and lets others fade into the background. Realising that there is no such a thing as *one* kind of complexity constitutive of and produced through an intervention might liberate researchers in thinking about and carrying out evaluation studies. Each form of complexity has its own consequences, and therefore using a specific definition of complexity (including leaving its definition open) is not an innocent choice that can be justified by pointing to the intervention itself. Rather, it is a choice with methodological, normative and political components and consequences (cf. Mol, 2002).

While Mol and Law’s (2002) writing on complexities (as well as that of Arribas-Ayllon *et al*., 2010) is a useful reminder to postpone judgments as to what complexity is, their notion of complexity is not analytically-privileged either. Indeed, Mol and Law’s understanding of complexity—that is, as mess that we should regard as (but is not necessarily) undefinable—accords with some biomedical actors’ own ‘complex’ accounts of this notion. This, we suggest, raises wider questions about the extent to which social scientists (like ourselves) might productively co-theorise with scientists and other actors about their terms of art (see, relatedly, Fitzgerald and Callard, 2014), as opposed to figuring the former community as necessarily having epistemological priority regarding the conceptualisation of biomedical concepts. This would also mean paying close attention to the temporal, affective and practical complexities involved in actual evaluation studies, an analysis that would get lost (or at least be less rich) were we to restrict the status of complexity to only being an actor’s, and not an analyst’s, category (Arribas-Ayllon *et al.*, 2010). Complexity is complicated, after all.

## Notes

1 While we draw mostly on STS-literatures in this article, we are aware that complexity is used in other disciplines as well. For example, it is a concept used in the natural sciences in order to grasp chaos and complexity in the physical world, including any observer effects on knowledge production (Prigogine and Stengers, 1984). It is also a concept used within theories on ‘self-organizing systems’, as for example used by Clark (1997) to theorise the relationship between the brain, the body and the outside world. This is probably not coincidental, and in fact might be related to a (anti-)grand narrative within contemporary society relating to complexity (where everything is seen as complex). However, in this paper we refrain from any comparisons outside of the subject matter we studied. Exactly because ontologies of complexity can be (and probably are) different across disciplines and therefore the interrelated practices of treating complexity as well (cf. Smith, 2005), arguing for a resemblance, we feel, would undermine our analysis of specific (and at times rather mundane) practices.

2 Further, we are also aware that as social scientists participating in the healthcare field, our own exposure to—and accumulation of—different kinds of capital in this realm has implications for our epistemic innocence when it comes to collecting and interpreting the data presented here (cf. Pickersgill, 2016).

3 Whereas ZonMw had commissioned national quality programmes and campaigns before, the programmes discussed here were among the first to include a formal evaluation running alongside the programme. Earlier programmes had been evaluated post hoc.

4 We would like to thank Kor Grit for helping us to articulate this argument.
